# Immobilization of *Acinetobacter* sp. A-1 and Applicability in Removal of Difenoconazole from Water–Sediment Systems

**DOI:** 10.3390/microorganisms13040802

**Published:** 2025-04-01

**Authors:** Feiyu Chen, Liping Wang, Yi Zhou, Jingyi Sui, Tianyue Wang, Jia Yang, Xiuming Cui, Ye Yang, Wenping Zhang

**Affiliations:** Key Laboratory of Sustainable Utilization of Panax Notoginseng Resources of Yunnan Province, Faculty of Life Science and Technology, Kunming University of Science and Technology, Kunming 650500, China; chenfeiyv2002@163.com (F.C.); lpwang0301@126.com (L.W.); zhouyi001016@163.com (Y.Z.); suijingyi29@163.com (J.S.); wangtianyue26@163.com (T.W.); jia091502@163.com (J.Y.); sanqi37@vip.sina.com (X.C.)

**Keywords:** difenoconazole, *Acinetobacter* sp., biodegradation, immobilization

## Abstract

Difenoconazole, as a systemic triazole fungicide, is a broad-spectrum, highly effective agent that has been widely used for controlling fungal diseases in 46 different crops (or crop categories), including rice, wheat, and corn. Due to the improper use of difenoconazole, concerns about its environmental residues and toxicity to non-target organisms have drawn significant attention from researchers. In response to this issue, this study aimed to isolate microbial strains capable of degrading difenoconazole from the environment. A novel difenoconazole-degrading strain, *Acinetobacter* sp. A-1, was screened and identified, demonstrating the ability to degrade 62.43% of 50 mg/L difenoconazole within seven days. Further optimization of the degradation conditions was conducted using single-factor experiments and response surface methodology experiments. The results showed that the optimal degradation conditions for strain A-1 were a difenoconazole concentration of 55.71 mg/L, a pH of 6.94, and an inoculation volume of 1.97%, achieving a degradation rate of 79.30%. Finally, strain A-1 was immobilized using sodium alginate, and its stability and bioremediation efficiency were evaluated. The results indicated that the immobilized strain A-1 exhibited high stability and significantly reduced the half-life of difenoconazole in the water–sediment contamination system. In the sterilized water–sediment system, the degradation rate of difenoconazole by the immobilized strain A-1 reached 65.26%. Overall, this study suggests that *Acinetobacter* sp. A-1 is a promising candidate for difenoconazole degradation, and immobilization technology can effectively enhance its removal efficiency in water–sediment systems.

## 1. Introduction

Difenoconazole (DIF) is a triazole fungicide commonly used to control fungal diseases affecting crops. It plays an important role in the field of agricultural production. It destroys the cell membrane by inhibiting the synthesis of ergosterol in fungal cells, thereby producing a bactericidal effect. It has been registered for the control of various diseases of cereals, vegetables, fruits, and herbs, including leaf spot, anthracnose, fusarium wilt, root rot, and powdery mildew [1]. With the acceleration of industrialization and the improvement of urbanization, the environmental pollution caused by the widespread use of pesticides cannot be ignored. Due to its wide range of applications and persistence, DIF has been detected in a variety of environmental matrices, including soil [2], water [3], and crops [4,5]. Pan et al. [2] found that DIF concentrations in more than 1% of soil samples in Northern China exceeded 0.1 mg/kg. A study has shown that after spraying DIF, the final residue on Rosa roxburghii was as high as 2.181 mg/kg, and the final residue in soil was as high as 2.406 mg/kg [4]. Even more worrisome is that a growing body of evidence indicates that DIF can negatively impact non-target species. One study found that exposure to 1.0 μg/L and 10.0 μg/L DIF inhibited gamete maturation and induced reproductive toxicity in zebrafish [6]. Additionally, it has been demonstrated that a high level of DIF can cause liver deformation, intestinal dysbiosis, and abnormal lipid metabolism in zebrafish [7]. It is therefore of great theoretical and practical importance to carry out systematic research into DIF degradation technology.

In recent years, with growing concern over pesticide residues, research and application of microbial degradation technology, an environmentally friendly treatment method, has increased significantly. The term ‘microbial degradation’ is used to denote a biodegradation process that employs the metabolic capacity of microorganisms to break down organic chemicals (e.g., pesticides, fungicides, and other natural pollutants), and this process is considered to be a significant method of removing pollutants from the environment [8]. With the continuous progress of science and technology, researchers have found that the use of natural microbial resources (including bacteria, fungi, and algae) can effectively metabolize pesticides or change their chemical structure, thereby accelerating the decomposition of pesticides and achieving the purification of the polluted environment [9,10]. Microorganisms have the advantages of strong tolerance and less secondary pollution, thus meeting the standards of sustainable development [11,12,13]. Nowadays, more and more researchers have begun to use microorganisms for pollution remediation. Thi et al. [14] reported the degradation of DIF by *Klebsiella* sp. (D5-2), *Citrobacter* sp. (D9-1), and *Pseudomonas* sp. (D10-3). It was determined that these three strains exhibited the capacity to completely degrade DIF (100 mg/L) within a 12-day period. Pinto et al. evaluated the biodegradability of DIF by fungi such as *Fusarium oxysporum*, *Aspergillus oryzae*, *Lentinula edodes*, *Penicillium brevicompactum*, and *Lecanicillium saksenae* [15]. The results showed that *L. edodes* is a fungus with high biodegradation potential for DIF. However, it is important to note that microbial degradation technology is not without its drawbacks. In the bioremediation of pesticide pollution, degrading bacteria may have nutrient deficiency and fierce competition with local bacteria, as well as the adverse effects of toxic substances in soil [16]. Therefore, how to effectively improve the treatment effect of microorganisms and expand their application range and pathway has become the focus of current research. The application of microbial immobilization technology can significantly improve both the survival rate and biological activity of degrading bacteria [17]. Microbial immobilization technology can not only maintain the improvement of microbial activity but also protect cells from external harmful factors [18]. Therefore, its application in the field of environmental remediation has become a global research hotspot. Microbial immobilization is a technique that confines free microorganisms within a specific carrier space through physical or chemical methods, thereby extending their life and maintaining cellular activity [19,20]. The choice of carrier material is one of the important factors affecting microbial immobilization [21,22]. Currently, the most widely used carriers can be classified into four main categories: inorganic, organic, composite, and novel carriers. Sodium alginate (SA) is one of the commonly used natural organic carriers. SA is a permeable, non-toxic, and transparent matrix, which is widely used for microbial immobilization due to its biocompatibility, biodegradability, and excellent film-forming properties [13,23]. SA offers a mild environment that supports microbial viability while protecting immobilized cells from extreme variations in chemical and physical conditions during immobilization [24,25]. Compared to free microorganisms, immobilized cells demonstrate multiple advantages, such as higher cellular density and metabolic activity per unit volume, minimized cell loss, accelerated reaction rates, facilitated recovery and reuse, and enhanced tolerance to environmental variations and toxic substances [13,18,26,27,28,29]. Therefore, immobilized microbial technology has gained widespread applications in environmental bioremediation. Wen et al. [30] conducted remediation experiments of sulfamethazine (SM2) contamination using immobilized strain H38. The findings revealed that 90% of SM2 was degraded in soil by the immobilized strain H38 within 12 days. Therefore, Xiang et al. [31] immobilized *Chryseobacterium* sp. H5 on novel polyvinyl alcohol/SA/biochar beads for bioremediation of thiamethoxam. The study demonstrated that the immobilized strain H5 removed approximately 90.47% of thiamethoxam within a 7-day period, with concurrent improvement in microbial tolerance to extreme conditions. Nazos and Ghanotakis [32] investigated the biodegradation of phenol by immobilized *Chlamydomonas reinhardtii*. It was observed that immobilized *C. reinhardtii* exhibited the capacity to remove up to 1300 μmol/L of phenol within 10 days.

The present study characterized the degradation properties of DIF using newly isolated DIF-degrading bacteria. Bacterial immobilization was achieved through an embedding method employing SA as the carrier material, followed by comprehensive evaluation of their stability and their bioremediation performance in water–sediment pollution systems. These results present a potential solution for the efficient remediation of DIF contamination in polluted environments, while providing a theoretical basis for evaluating both the effectiveness and the ecological safety of immobilized microbial-based DIF remediation strategies.

## 2. Materials and Methods

### 2.1. Isolation, Screening, and Identification of DIF-Degrading Bacteria

DIF-degrading bacterial strains were isolated from long-term pesticide-contaminated soils in Chinese herbal medicine plantations in Kunming, Yunnan Province. The isolation and enrichment of DIF-degrading bacteria followed the method described by Chen et al. [33]. Initial cultivation was performed in MSM containing 50 mg/L DIF at 30 °C, with agitation at 200 rpm for 7 days. Subsequently, the cultures were subjected to enrichment in MSM with 100 mg/L DIF using a 2% inoculum, followed by sequential transfers to media with progressively higher DIF concentrations to promote microbial domestication. The enriched culture was serially diluted and spread onto solid Luria-Bertani (LB) medium. The grown strain was isolated and subjected to two successive rounds of purification to obtain a single colony. The single colony was designated as strain A-1. Strain A-1 was activated and inoculated into MSM containing 50 mg/L DIF for 7 days. The residual DIF in the medium was extracted using ethyl acetate, and its concentration was determined via high-performance liquid chromatography (HPLC). The specific implementation followed the method described by Zhou et al. [34].

Morphological analysis of strain A-1 was performed using a transmission electron microscope (L208-3M180 AOSVI, Shenzhen, China) and a scanning electron microscope (SEM; Regulus 8100, Hitachi, Japan) [35]. Molecular identification of strain A-1 was carried out through polymerase chain reaction (PCR) amplification of the 16S rRNA gene. The amplified sequence was analyzed using BLAST (1.4.0) in the GenBank database, and phylogenetic reconstruction was performed using MEGA X 11.0.11 to identify homologous sequences and analyze evolutionary relationships.

### 2.2. Determination of Growth and Degradation Capacity of Free DIF-Degrading Bacteria

To investigate the relationship between the growth of strain A-1 and DIF degradation, the strain was initially cultured in LB medium for activation. Subsequently, the activated culture was inoculated at 5% (*v*/*v*) into 20 mL of minimum salt medium (MSM) containing 50 mg/L DIF. The cultures were incubated at 30 °C, with agitation at 200 rpm for 7 days, during which daily sampling was performed to monitor both bacterial growth and DIF degradation. Bacterial growth was monitored by measuring OD_600_. The residual DIF content was determined through quantitative analysis using HPLC. Each sample was analyzed in triplicate. Samples without strain A-1 were included as controls.

### 2.3. Optimization of Culture Conditions for DIF Degradation by Strain A-1

The effects of various culture conditions on DIF degradation efficiency by strain A-1 were systematically investigated through single-factor optimization, followed by response surface methodology (RSM). Strain A-1 was inoculated into MSM supplemented with DIF and cultured at 30 °C, with agitation at 200 rpm for 7 days. The residual DIF content was determined through quantitative analysis using HPLC. The single-factor conditions were set as follows: pH (5, 6, 7, 8, and 9), inoculum size (1%, 2%, 5%, 10%, and 15% *v*/*v*), and substrate concentration (30, 40, 50, 60, and 70 mg/L). Each sample was replicated in triplicate.

Based on the single-factor experimental results, three critical parameters—pH, inoculum size, and substrate concentration—were selected for optimization, with the DIF degradation rate as the response variable. A Box–Behnken experimental design was employed to optimize the response surface using a three-factor, three-level approach, as detailed in Table 1. Each sample was replicated in triplicate.

### 2.4. Degradation Kinetics Assay

To characterize the degradation kinetics of strain A-1 at varying DIF concentrations, kinetic parameters were determined through nonlinear regression analysis. The kinetic data describing strain A-1-mediated DIF degradation under varying substrate concentrations, as presented in Section 2.3, were analyzed using the Andrews equation with Origin 2022. The relationship between *S* (DIF concentration, mg/L) and *q* (specific degradation rate, d^−1^) was plotted. According to the Andrews equation, the experimental values were fitted using a nonlinear least squares curve, and the optimal initial concentration of DIF degradation by strain A-1 was calculated [36]. The equation is expressed as follows:

(1) $$q=\frac{q_{max}S}{S+K_{S}+\frac{S^{2}}{K_{i}}},$$

where *S* is the inhibitor concentration (mg/L), *q* is specific degradation rate (d^−1^), *q_max_* is the maximum specific degradation rate (d^−1^), *K_s_* is the half-saturation constant (mg/L), and *K_i_* is the substrate inhibition constant (mg/L).

### 2.5. Immobilization of Strain A-1 and Degradation Assays

Strain A-1 was cultured in liquid LB medium for 24 h at 30 °C, with agitation at 200 rpm. Subsequently, strain A-1 cells were harvested by centrifugation at 4 °C and 4000 rpm, washed twice with sterile physiological saline (0.9% NaCl), and resuspended in sterile saline solution. Five milliliters of bacterial suspension was thoroughly mixed with 45 mL of 3% (*v*/*v*) SA solution, under constant agitation at 200 rpm for 30 min. The mixture was manually injected into a 4% (*w*/*v*) CaCl_2_ solution using a sterile syringe, followed by cross-linking at 4 °C for 24 h to form immobilized pellets. The immobilization effect of strain A-1 was observed using SEM. To enhance the immobilization efficiency of strain A-1, immobilization parameters were optimized using a single-factor experimental design. The effects of different SA concentrations (2.0, 2.5, 3.0, 3.5, and 4.0% *w*/*v*) and CaCl_2_ concentrations (3.0, 3.5, 4.0, 4.5, and 5% *w*/*v*) on the degradation of DIF by immobilized strain A-1 were determined. Additionally, a comparative analysis of DIF degradation efficiency between free and immobilized forms of strain A-1 was performed in MSM. Each sample was replicated in triplicate. Samples without immobilized strain A-1 and bacterial suspension were included as controls.

### 2.6. Effect of Culture Conditions on the DIF Degradation Ability of Immobilized Strain A-1

The optimal degradation conditions for immobilized strain A-1 were determined by investigating the effects of temperature (20, 25, 30, 35, and 40 °C), pH (5, 6, 7, 8, and 9), and substrate concentration (30, 40, 50, 60, and 70 mg/L) on DIF degradation efficiency, following the experimental protocol described in Section 2.3. The residual DIF content was determined through quantitative analysis using HPLC. Each sample was replicated in triplicate.

### 2.7. Degradation Ability of Immobilized and Free Strain A-1 in Water–Sediment System

The degradation potential of strain A-1 in water–sediment contaminated systems was evaluated using the protocol developed by Li et al. [37]. A 50 mL water–sediment system (WSS) was prepared by combining 10% (*w*/*v*) soil with 90% (*v*/*v*) sterile water. Soil samples were collected from the 0–20 cm soil layer of farmland with no history of pesticide application. Soil samples were sieved (2 mm) for further analysis. Immobilized and free bacterial cells were inoculated into sterilized (121 °C for 30 min) and nonsterilized WSSs, respectively, at an inoculum density of 1.4 × 10^7^ CFU/mL. The final DIF concentration in the WSS was adjusted to 50 mg/L. The bioremediation system was incubated at 30 °C, with agitation at 200 rpm for 5 days, and with uninoculated treatments serving as controls. The residual DIF in the WSS was quantified using HPLC.

The degradation kinetics of DIF by both immobilized and free strain A-1 were analyzed using a first-order kinetic model to determine the corresponding kinetic parameters. The first-order kinetic fitting was performed using Equation (2), and the half-life (*t*_1/2_) of DIF was calculated using Equation (3) [38]:

(2) $$C_{t}=C_{0}\times e^{-kt},$$

(3) $$t_{1/2}=\frac{ln\;2}{k}$$

where *C*_0_ is the initial concentration of DIF (mg/L), *C_t_* is the DIF content at time *t*, *k* is the degradation constant (d^−1^), and *t* is the degradation time (d). *ln* 2 is the natural logarithm of 2, and *t*_1/2_ is the half-life of DIF (d).

## 3. Results and Discussion

### 3.1. Isolation, Screening, and Identification of Strain A-1

**Figure 1:**
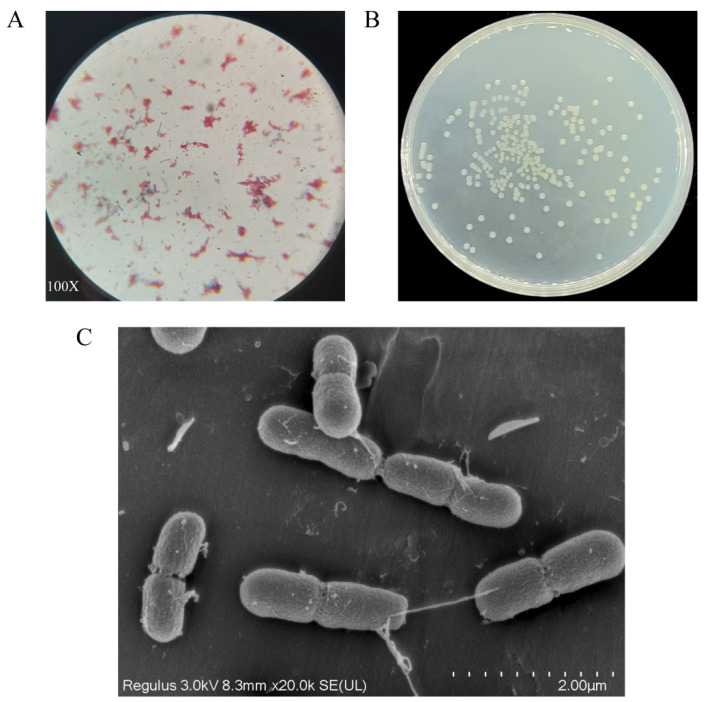
Gram-staining results of A-1 (**A**). The colony morphology of A-1 (**B**). SEM image of A-1 (**C**).

**Figure 2:**
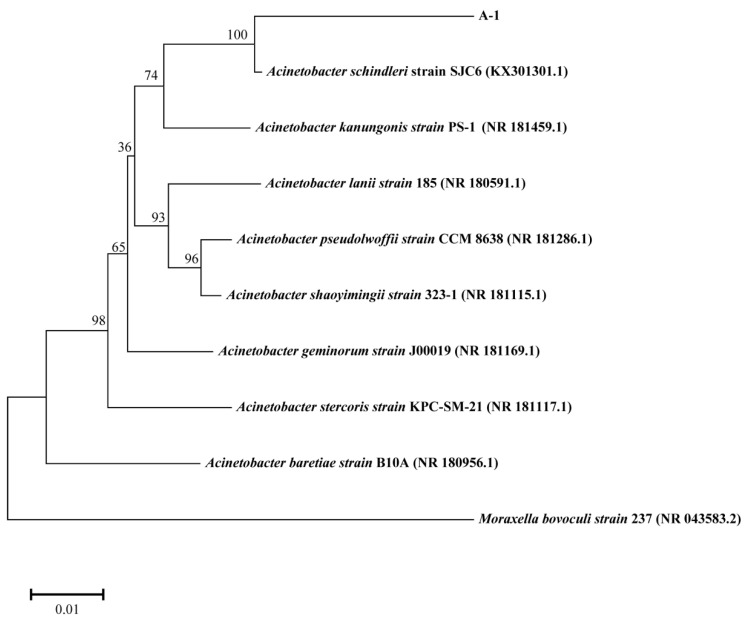
Phylogenetic tree based on concatenated nucleotide sequences of 16S rDNA of A-1 and other representative strains. Consensus sequences of every gene from related strains were aligned with ClustalW and trimmed to the same sizes. Bootstrap values after 1000 replicates are expressed as percentages. The scale bar denotes nucleotide substitutions per site.

Through sequential gradient domestication and enrichment culture, we isolated a novel bacterial strain, designated A-1, from pesticide-contaminated soil samples. This strain demonstrated the ability to utilize DIF as its sole carbon and energy source. Strain A-1’s DIF degradation capability was evaluated in minimal salt medium (MSM) containing 50 mg/L DIF, showing 62.43% degradation efficiency after 7 days of incubation. Microscopic and macroscopic characterization identified strain A-1 as a Gram-negative bacterium (Figure 1A). Colonies exhibited circular morphology with light yellow pigmentation and well-defined, smooth margins. Colonies displayed moist, shiny, and viscous surfaces with central elevation and opacity (Figure 1B). Scanning electron microscopy (SEM) revealed that strain A-1 possesses a rod-shaped morphology (Figure 1C). The physiological and biochemical properties of strain A-1 are summarized in Supplementary Table S1. The 16S ribosomal DNA (rDNA) sequence of strain A-1 was amplified using universal primers 27F and 1492R, followed by BLASTn analysis against the NCBI GenBank database for phylogenetic comparison. Comparative sequence analysis revealed 96% similarity between the 16S rDNA sequence of strain A-1 and that of several Acinetobacter species. Phylogenetic analysis using the neighbor-joining method with 1000 bootstrap replicates demonstrated that strain A-1 forms a distinct clade with *Acinetobacter schindleri* SJC6 (GeneBank Number: KX301301.1), as shown in Figure 2. Polyphasic taxonomic analysis, combining morphological, physiological, biochemical characteristics, and 16S rDNA sequence data, enabled the preliminary classification of strain A-1 within the genus *Acinetobacter*. The strain was deposited in the Guangdong Microbial Culture Collection Center (GDMCC), with the accession number GDMCC No. 64727. Strain A-1 exhibited significantly higher degradation efficiency for 50 mg/L DIF compared to *Phyllobacterium* sp. T-1 (lower than 30%) and *Aeromonas* sp. T-2 (lower than 30%) [39]. Notably, *Acinetobacter* spp. have been seldom documented as efficient degraders of DIF in the literature.

### 3.2. The Strain Growth and Degradation Characteristics of Strain A-1

**Figure 3:**
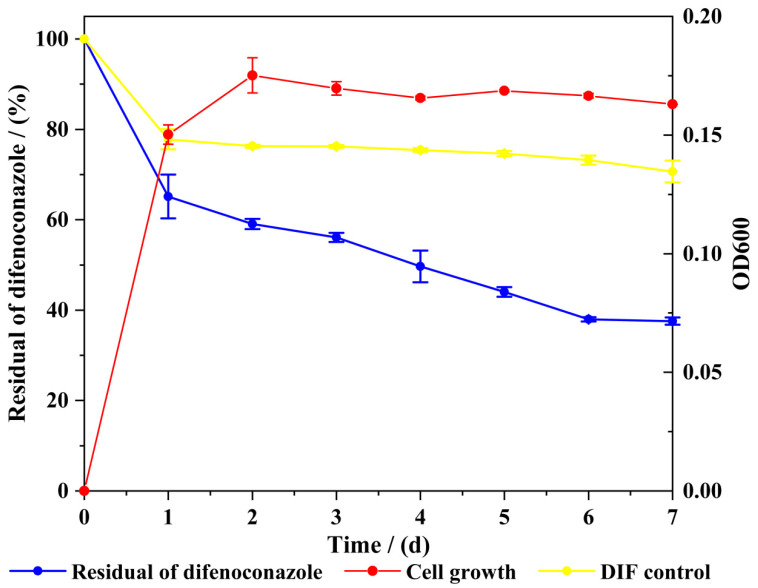
Degradation of difenoconazole and cell growth of strain A-1.

To examine the relationship between bacterial growth dynamics and DIF degradation capacity, strain A-1 was monitored for both growth kinetics and degradation efficiency throughout the process, as shown in Figure 3. Strain A-1 efficiently degraded DIF, utilizing it as the sole carbon source and converting it into essential metabolites to support growth and metabolic activities. Figure 3 demonstrates a positive correlation between DIF degradation efficiency and bacterial growth. In DIF-containing medium, strain A-1 showed a short lag phase and entered exponential growth within 24 h. During exponential growth (24–48 h), rapid bacterial proliferation coincided with maximum DIF degradation rates. Upon reaching stationary phase at 48 h, DIF degradation stabilized, followed by transition to the decline phase. After 7 days (168 h) of cultivation, DIF degradation efficiency reached 62.43%. In the abiotic control, natural DIF degradation after 7 days was below 30%.

### 3.3. Effects of Culture Conditions on the Removal of DIF by Strain A-1

**Figure 4:**
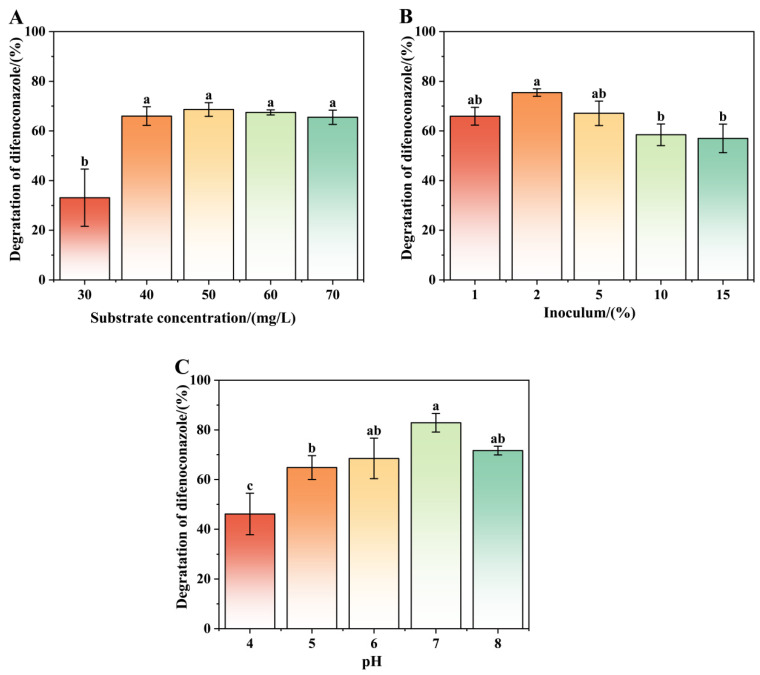
Effects of culture conditions on the degradation characteristics of strain A-1 difenoconazole: substrate concentration of difenoconazole (**A**), inoculum (**B**), and pH (**C**). Bars represent the means of triplicate identical replicates (*N* = 3); error bars indicate the standard deviation of the means. Different letters indicate significant differences (*p* < 0.05) between treatments.

The effects of three key parameters—substrate concentration, inoculum size, and Ph—on DIF degradation by strain A-1 are systematically presented in Figure 4. DIF degradation efficiency showed significant variation across experimental conditions, ranging from 20.89% to 85.76%, as detailed in Supplementary Tables S2–S4. Strain A-1 displayed concentration-dependent DIF degradation efficiency across initial concentrations of 30 to 70 mg/L, as shown in Figure 4A. As shown in Figure 4A, strain A-1’s DIF degradation efficiency increased with substrate concentration up to 50 mg/L. The maximum degradation efficiency of 68.63% was achieved at an optimal DIF concentration of 50 mg/L. At 30 mg/L DIF concentration, the degradation efficiency was significantly lower due to substrate limitation, restricting bacterial growth and metabolic activity. Above the optimal concentration (50 mg/L), DIF degradation efficiency gradually decreased, indicating substrate inhibition that affected both bacterial growth and enzymatic activity. This concentration-dependent inhibition pattern is consistent with previous studies on microbial degradation of similar compounds. For example, Chen et al. [39] observed that the degradation rate of DIF gradually decreased with increasing initial concentrations (20–100 mg/L). Additionally, Huang et al. [40] reported that the degradation rate of quinoline acid (QNC) by *Cellulosimicrobium cellulans* strain D gradually increased within the range of 20–50 mg/L, but it significantly decreased when the concentration exceeded 50 mg/L.

Figure 4B illustrates the degradation efficiency of DIF (50 mg/L) by strain A-1 under different inoculation amounts. As the inoculum size increased, the DIF degradation rate initially increased and subsequently decreased. At inoculum sizes of 1%, 5%, 10%, and 15%, the DIF degradation rates were consistently lower than that observed at 2% inoculum. These results demonstrate that an optimal inoculum size significantly enhances DIF decomposition efficiency. As the inoculum size increased from 1% to 2%, the DIF degradation rate exhibited a gradual increase. The maximum degradation rate of 75.43% was achieved at 2% inoculum after 7 days of incubation. At 5% inoculum, the degradation rate showed no significant improvement, likely due to substrate limitation caused by excessive bacterial growth, which restricted both cellular proliferation and metabolic activity. At low inoculum levels, the extended growth cycle and reduced cellular activity of the strain significantly prolong the time required to achieve optimal degradation efficiency [41]. Conversely, high inoculum levels lead to shortened growth cycles and diminished cellular vitality due to substrate limitation, which restricts both growth and metabolic activities [42].

The DIF degradation efficiency of strain A-1 across a pH range of 4 to 8 is presented in Figure 4C. Maximum degradation efficiency (82.88%) was achieved at pH 7. At pH values of 4, 5, 6, and 8, degradation efficiencies ranged from 46% to 72%. In 50 mg/L DIF medium, degradation efficiency showed a bell-shaped response to pH, increasing from pH 4 to 7 and then decreasing. These results indicate that strain A-1 can effectively degrade DIF across a wide pH range (4–8). The optimal pH for DIF degradation was 7, although the strain maintained significant degradation activity in both weakly acidic (pH 5–6) and weakly alkaline (pH 8) conditions. However, at pH 4, degradation efficiency decreased significantly, likely due to inhibited cellular growth and reduced enzymatic activity, limiting the strain’s degradation capacity [38].

The coded values and corresponding response results from the Box–Behnken experimental design are presented in Table 2. Analysis revealed that DIF degradation efficiency by strain A-1 varied significantly (47.90% to 78.61%) across different culture conditions, demonstrating the substantial impact of these parameters on degradation performance. Using Design-Expert 13.0 software, multiple regression analysis of the experimental data generated a quadratic polynomial regression model for DIF degradation, incorporating three key factors: pH (A), substrate concentration (B), and inoculum size (C):

Y = 76.08 − 0.3750 × A + 8.66 × B − 0.9125 × C − 1.30 × AB + 0.9000 × AC + 0.7250 × BC − 10.20 × A^2^ − 7.63 × B^2^ − 9.68 × C^2^ (4)

where Y is the degradation rate of DIF, A is pH, B is substrate concentration, and C is inoculum. The experimental results were analyzed using analysis of variance (ANOVA), with the detailed results presented in Table 3. The model demonstrated high statistical significance, with an F-value of 85.78, and *p*-value < 0.0001. The high coefficient of determination (*R*^2^ = 0.9827) and adjusted *R*^2^ (*R_Adj_*^2^ = 0.9795) indicate excellent agreement between experimental and predicted DIF degradation rates. The model accounts for 98.27% of the total variation in response values. The lack-of-fit test showed an F-value of 0.4229 (*p* = 0.7473), confirming its non-significance (*p* > 0.05). These results confirm that the model accurately represents the experimental system and reliably predicts the effects of culture conditions on DIF degradation by strain A-1. The regression model revealed the following factor-influence hierarchy: substrate concentration > inoculum size > pH, with all factors showing positive effects. The quadratic terms (A^2^, B^2^, and C^2^) significantly influenced DIF degradation.

**Figure 5:**
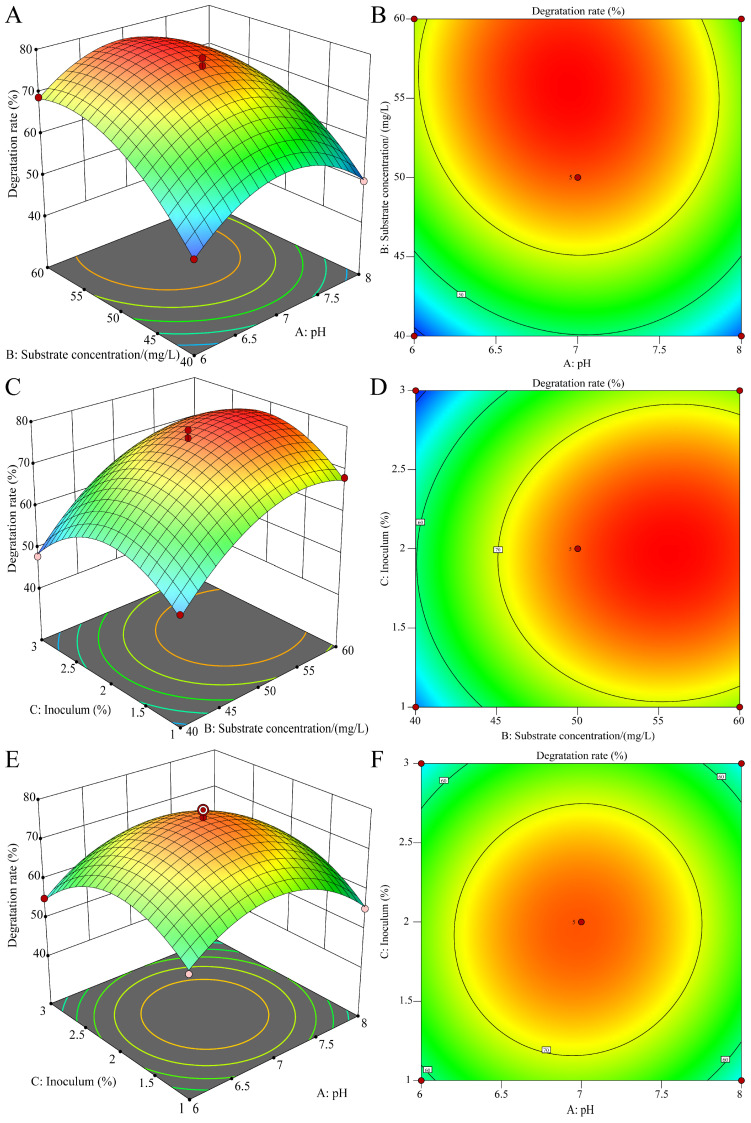
Optimization of difenoconazole degradation with strain A-1 using response surface methodology. Three-dimensional response surface (**A**) and contour (**B**) plots illustrating the interactive effects of pH and substrate concentration on difenoconazole degradation; three-dimensional response surface (**C**) and contour (**D**) plots illustrating the interactive effects of inoculum and substrate concentration on difenoconazole degradation; and three-dimensional response surface (**E**) and contour (**F**) plots illustrating the interactive effects of pH and inoculum on difenoconazole degradation.

Response surface and contour plots were generated using Design-Expert 13.0 based on the Box–Behnken design regression model, as presented in Figure 5. The interaction effects were evaluated through response surface slope analysis and contour line morphology. Gentle slopes with circular contours indicated weak interactions, while steep slopes with elliptical contours suggested strong interactions. The software analysis identified optimal conditions as pH 6.94, 55.71 mg/L substrate concentration, and 1.97% inoculum, predicting a maximum degradation efficiency of 79.30%. For practical implementation, the conditions were adjusted to pH 7.0, 56 mg/L substrate concentration, and 2.0% inoculum. Triplicate validation experiments achieved 79.78% degradation efficiency, demonstrating excellent agreement with model predictions.

### 3.4. Kinetic Analysis of DIF Degradation

**Figure 6:**
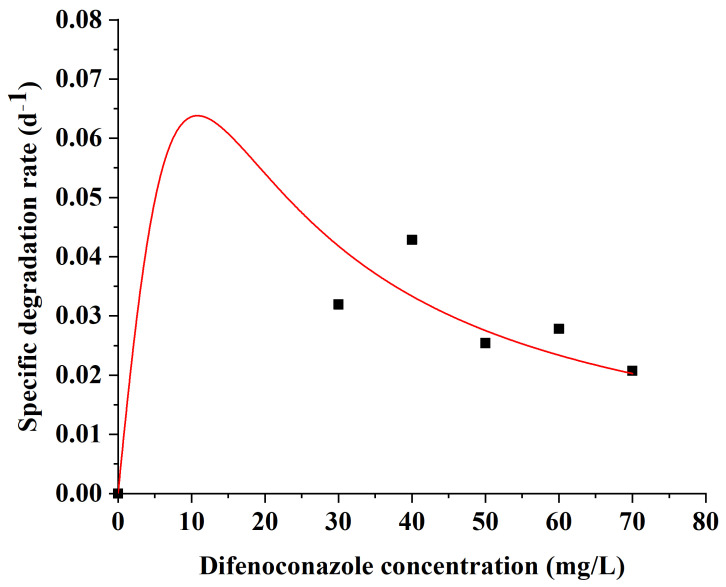
Relationship between specific degradation rate and initial difenoconazole concentration.

Figure 6 presents the degradation kinetics of strain A-1 for DIF at different initial concentrations. Nonlinear regression analysis using Origin 2022 (9.9) yielded the following kinetic parameters: *q_max_* = 0.89 d^−1^, *K_s_* = 70 mg/L, and *K_i_* = 1.67 mg/L. The model showed a good fit to the experimental data, with a coefficient of determination (*R*^2^) of 0.79. Mathematical differentiation identified 10.81 mg/L as the optimal initial DIF concentration for maximum specific degradation rate. Strain A-1 effectively utilized DIF as a sole carbon source across a wide concentration range. The broad operational range of DIF degradation rates highlights strain A-1’s potential for large-scale bioremediation applications. The kinetic analysis provides crucial insights for optimizing strain A-1’s practical application in DIF bioremediation.

### 3.5. The DIF Degradation Capacity of Immobilized Strain A-1

**Figure 7:**
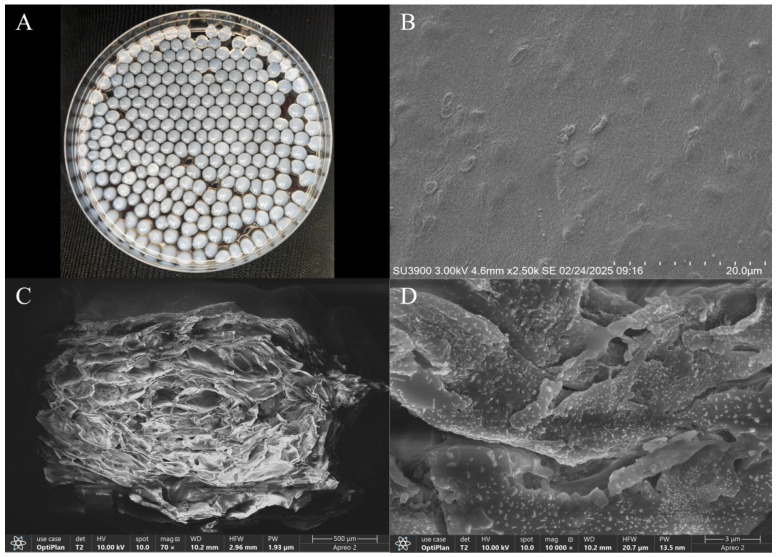
The surface morphology and microstructure of immobilized strain A-1: (**A**) appearance, (**B**) surface morphology (2500×), (**C**) microstructure (70×), and (**D**) microstructure (10,000×).

Figure 7 presents the surface morphology and internal microstructure of immobilized strain A-1. The embedding method produced immobilized strain A-1 with excellent sphericity, uniform size distribution, and no tailing, as shown in Figure 7A, meeting all requirements for subsequent analysis. Figure 7B reveals a relatively smooth surface and compact structure of the immobilized strain A-1. Surface pores (approximately 2 μm in diameter) facilitate DIF diffusion into the beads, enhancing substrate–microbe interactions. Bacterial cells were predominantly located within the beads, with minimal surface immobilization. The internal microstructure, shown in Figure 7C,D, demonstrates high porosity and a robust internal framework that supports strain A-1 growth. This structure ensures efficient DIF diffusion and metabolite release during bacterial growth. The framework structure significantly increases the internal surface area, enhancing microbial immobilization efficiency.

**Figure 8:**
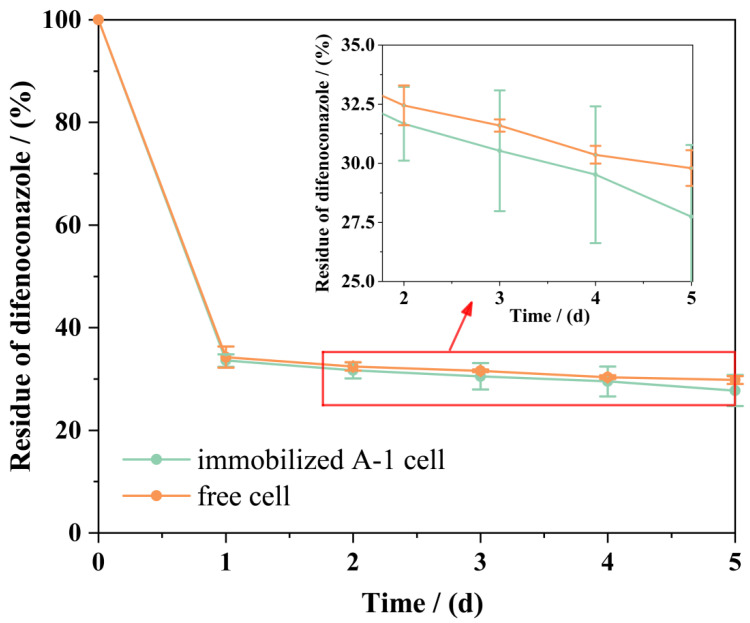
Performance of immobilized strain A-1 degrading difenoconazole. Residue of difenoconazole in difenoconazole degradation by free strain A-1 and immobilized strain A-1. Bars represent the means of triplicate identical replicates (*N* = 3); error bars indicate the standard deviation of the means.

Figure 8 compares the DIF degradation performance of immobilized strain A-1 and free cells in MSM. Both immobilized strain A-1 and free cells demonstrated effective DIF removal from MSM. Immobilized strain A-1 showed marginally higher DIF removal efficiency compared to free cells. Within 24 h, immobilized strain A-1 achieved 66.41% DIF degradation, comparable to free cells’ efficiency (65.74%). Degradation efficiency progressively increased to 72.23% after 120 h. Free cells consistently showed lower degradation rates, reaching 70.20% after 120 h. The enhanced degradation efficiency of immobilized strain A-1 may be attributed to the protective encapsulation provided by SA, which improves the strain’s tolerance to DIF and maintains its metabolic activity. Notably, SA immobilization preserves cellular integrity without inducing significant physicochemical changes during encapsulation [43], unlike alternative immobilization matrices. The resultant alginate beads retain optimal permeability and structural transparency, enabling efficient passive diffusion and adsorption of DIF molecules into the polymeric network.

### 3.6. Components Optimization of Immobilized Conditions

**Figure 9:**
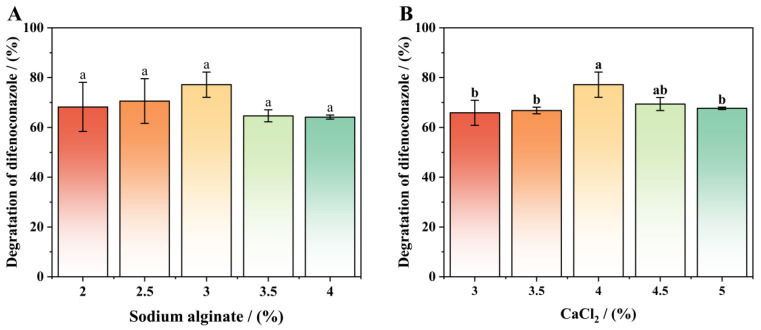
Optimization of immobilized conditions: sodium alginate (**A**) or CaCl_2_ (**B**). Bars represent the means of triplicate identical replicates (*N* = 3); error bars indicate the standard deviation of the means. Different letters indicate significant differences (*p* < 0.05) between treatments.

Figure 9A,B illustrate the effects of SA and CaCl_2_ concentrations on DIF degradation by immobilized strain A-1. Immobilized strain A-1 demonstrated significant DIF degradation capacity across various SA concentrations. Figure 9A shows a bell-shaped response of degradation efficiency to increasing SA concentration. Optimal degradation efficiency (77.26%) was achieved at 3% (*w*/*v*) SA, where the beads maintained perfect sphericity. At SA concentrations below 3%, the immobilized strain A-1 exhibited tailing effects, reduced viscosity, and inadequate mechanical strength, which compromised bacterial immobilization efficiency, leading to fewer immobilized cells and consequently lower DIF degradation rates [44,45]. However, higher SA concentrations led to slower bead formation, irregular shapes, and increased solution viscosity and mechanical strength, which impaired mass transfer, reduced cellular metabolic activity, and ultimately decreased DIF degradation efficiency [44,45,46].

Figure 9B shows the DIF degradation efficiency of immobilized strain A-1 across varying CaCl_2_ concentrations. DIF degradation efficiency exhibited a bell-shaped response to increasing CaCl_2_ concentration. Compared to the optimal 4% CaCl_2_ concentration, significantly lower degradation efficiencies were observed at both suboptimal (3%, 3.5%) and supraoptimal (4.5%, 5%) concentrations. From 3% to 4% CaCl_2_, DIF degradation efficiency increased progressively with concentration. Maximum degradation efficiency (77.18%) was achieved at 4% CaCl_2_ after 7 days of incubation. Above the optimal concentration (4.5% CaCl_2_), DIF degradation efficiency decreased significantly. Gel strength showed a positive correlation with CaCl_2_ concentration [45]. At low CaCl_2_ concentrations, the immobilized beads exhibit weak structural stability, increasing susceptibility to environmental stresses and resulting in cell loss or reduced metabolic activity. Conversely, high CaCl_2_ concentrations produce excessively compact bead structures that impede DIF uptake and metabolite excretion, ultimately reducing cellular activity and stability.

### 3.7. Determination of Environmental Adaptability of Immobilized Strain A-1

**Figure 10:**
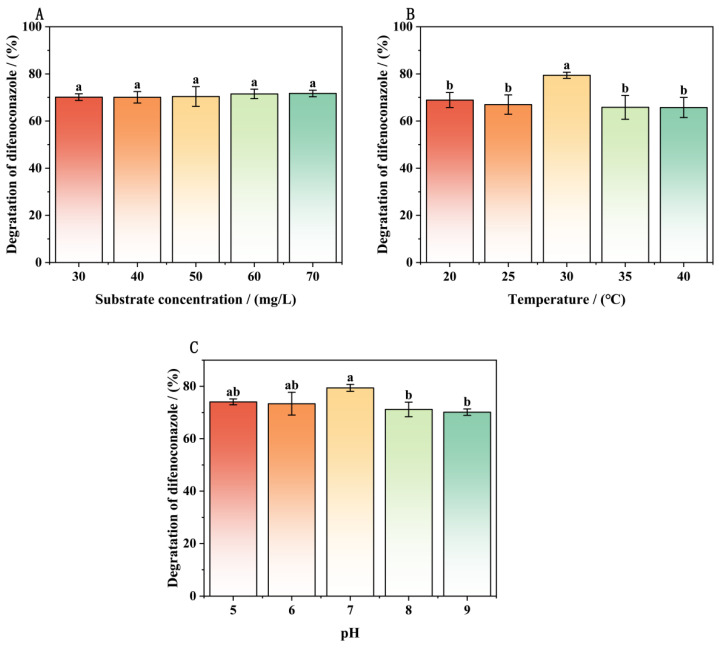
Determination of environmental adaptability of immobilized strain A-1 (3d): substrate concentration of difenoconazole (**A**), temperature (**B**), and pH (**C**). Bars represent the means of triplicate identical replicates (*N* = 3); error bars indicate the standard deviation of the means. Different letters indicate significant differences (*p* < 0.05) between treatments.

To assess environmental adaptability, we evaluated the effects of substrate concentration, temperature, and pH on DIF degradation by immobilized strain A-1. Figure 10 demonstrates the effective DIF removal by immobilized strain A-1 across various environmental conditions. Immobilized strain A-1 showed significantly enhanced stability and environmental adaptability compared to free cells. Immobilized strain A-1 maintained high DIF degradation efficiency across a wide concentration range (Figure 10A). DIF degradation efficiency by immobilized strain A-1 increased progressively with substrate concentration. Maximum degradation efficiency (71.70%) was achieved at 70 mg/L substrate concentration. However, as shown in Figure 10A, substrate concentration showed limited influence on degradation efficiency, with only 1.47% variation between maximum and minimum values. All treatment groups achieved >70% DIF degradation within 3 days, showing significant improvement over free cells.

Figure 10B presents the temperature-dependent DIF degradation efficiency of immobilized strain A-1. Compared to the optimal 30 °C, lower degradation efficiencies (65–70%) were observed at both suboptimal (20 °C and 25 °C) and supraoptimal (35 °C and 40 °C) temperatures. Maximum degradation efficiency (79.43%) was achieved at the optimal temperature of 30 °C. These results demonstrate that immobilized strain A-1 maintains effective DIF degradation across a broad temperature range (20–40 °C). The optimal temperature for DIF degradation was determined to be 30 °C. Immobilization enabled strain A-1 to maintain significant degradation activity at both low and high temperatures.

Figure 10C illustrates the pH-dependent DIF degradation performance of immobilized strain A-1. The degradation efficiency exhibited a bell-shaped response to pH, increasing from pH 5 to 7 and then decreasing. Maximum degradation efficiency (79.43%) was achieved at the optimal pH of 7. At pH values of 5, 6, 8, and 9, degradation efficiencies remained high (70–74%). Compared to free cells, immobilized strain A-1 maintained the same optimal pH but showed enhanced degradation capacity. Immobilized strain A-1 maintained effective degradation in both weakly acidic (pH 5–6) and weakly alkaline (pH 8–9) conditions. These results demonstrate that immobilized strain A-1 can effectively degrade DIF across a broad pH range (5–9).

### 3.8. Comparison of the Bioremediation Effects of Free and Immobilized Strain A-1

**Figure 11:**
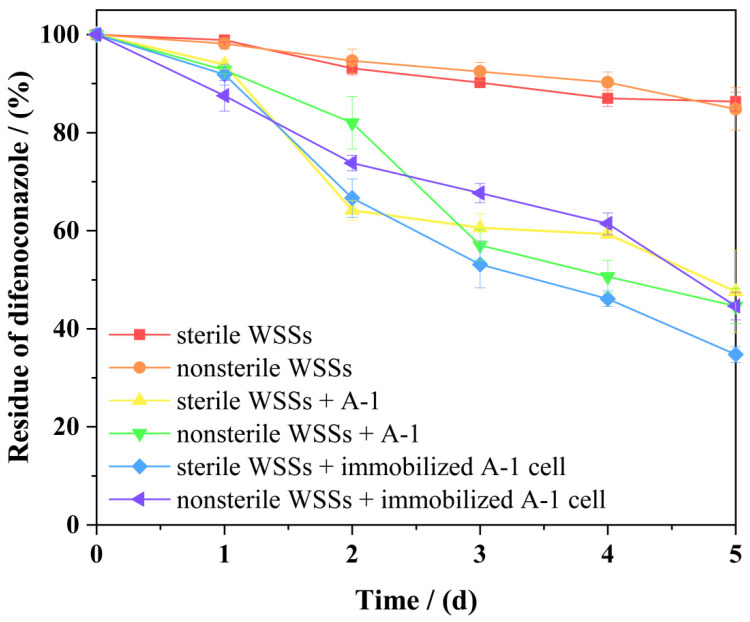
Comparison of the bioremediation effects of free and immobilized strain A-1. Bars represent the means of triplicate identical replicates (*N* = 3); error bars indicate the standard deviation of the means.

The bioremediation efficiency of free and immobilized cells was evaluated using simulated WSSs. The half-life (*t*_1/2_) of DIF in each WSS was calculated using first-order kinetics (Equations (2) and (3)), with results summarized in Table 4. Control groups showed *t*_1/2_ values of 21.07 and 22.06 days. Treatment groups exhibited significantly shorter *t*_1/2_ values: 3.91 and 4.71 days for free cells and 3.20 and 4.68 days for immobilized cells. The *t*_1/2_ in non-inoculated WSS was 5.00- and 5.47-fold longer than in free and immobilized cell treatments, respectively. These results demonstrate strain A-1’s significant capacity to accelerate DIF degradation in WSSs. This discovery aligns with the findings of Xiong et al. [47] and Yu et al. [48]. Figure 11 shows that immobilized strain A-1 achieved higher DIF degradation rates in WSS than free cells, demonstrating that immobilization enhances and stabilizes the strain’s degradation capacity in complex environments. Regardless of the inoculation method (free or immobilized cells), DIF half-life was consistently shorter in sterilized than nonsterilized WSS. This finding contrasts with the observations reported by Zhou et al. [34]. These results suggest potential antagonistic interactions between strain A-1 and indigenous microorganisms in the WSS, possibly affecting DIF metabolism.

## 4. Conclusions

In this study, we isolated a newly discovered DIF-degrading bacterium, *Acinetobacter* sp. A-1, capable of using DIF as its sole carbon and energy source. Strain A-1 was immobilized using sodium alginate as a carrier through an embedding method to produce an immobilized bacterial agent. The immobilized bacterial agent demonstrated a strong ability to degrade DIF over a broad range of DIF concentrations, temperatures, and pH levels, suggesting that the immobilized cells had improved environmental adaptability. Furthermore, compared with free cells, immobilized A-1 cells exhibited greater in situ biodegradation capacity in a water–sediment pollution system. This study suggests that immobilized strain A-1 offers a safe and effective bioremediation approach, presenting a promising new strategy for the environmental biodegradation of DIF. This study is nevertheless limited by incomplete genomic characterization of the isolate, unresolved molecular mechanisms underlying DIF degradation, and unverified long-term stability of the immobilized-cell formulation. Future studies should employ integrated proteomic and genomic approaches to identify key enzymes and genes involved in DIF degradation. This mechanistic understanding will facilitate the development of genetically engineered strains for enhanced bioremediation. Furthermore, field-scale validation is required to assess the environmental robustness of strain A-1 and optimize its application parameters for practical DIF bioremediation.

## Figures and Tables

**Table 1:** Box–Behnken design factor levels.

Factor	Name	−1	0	1
A	pH	6	7	8
B	Substrate concentration/(mg/L)	40	50	60
C	Inoculum/(%)	1	2	3

**Table 2:** Response surface test design and results.

Run Order	Factor			Degradation Rate/(%)
	A: pH	B: Substrate Concentration/(mg/L)	C: Inoculum/(%)	
1	7	50	2	78.61
2	7	60	3	66.50
3	8	50	3	56.70
4	7	50	2	75.48
5	7	60	1	68.20
6	7	40	3	47.90
7	8	60	2	65.10
8	6	50	1	57.50
9	8	50	1	55.40
10	6	60	2	68.90
11	7	50	2	75.71
12	7	40	1	52.50
13	6	40	2	48.80
14	7	50	2	76.78
15	6	50	3	55.20
16	7	50	2	73.81
17	8	40	2	50.20

**Table 3:** Quadratic polynomial regression model and significance test results.

Source	Sum of Squares	df	Mean Square	F-Value	*p*-Value	Significance
Model	1821.67	9	202.41	85.78	<0.0001	significant
A—pH	1.13	1	1.13	0.4767	0.5121	
B—substrate concentration/(mg/L)	600.31	1	600.31	254.40	<0.0001	
C—inoculum	6.66	1	6.66	2.82	0.1368	
AB	6.76	1	6.76	2.86	0.1344	
AC	3.24	1	3.24	1.37	0.2796	
BC	2.10	1	2.10	0.8910	0.3766	
A^2^	438.19	1	438.19	185.70	<0.0001	
B^2^	244.90	1	244.90	103.78	<0.0001	
C^2^	394.25	1	394.25	167.07	<0.0001	
Residual	16.52	7	2.36			
Lack of Fit	3.98	3	1.33	0.4229	0.7473	not significant
Pure error	12.54	4	3.14			
Cor total	1838.19	16				

Note: *R*^2^ = 0.9910; adjusted *R*^2^ = 0.9795; predicted *R*^2^ = 0.9547; C.V.%, 2.43; adeq precision, 23.4287.

**Table 4:** Kinetic parameters for the degradation of difenoconazole by free cells and immobilized A-1 cell in sterile and nonsterile WSSs.

Treatments	Regression Equation	*k*/(Day^−1^)	*R* ^2^	*t*_1/2_/(Days)
Sterile WSSs	*C_t_* = 50.18334e^−0.0329t^	0.0329	0.95996	21.07
Nonsterile WSSs	*C_t_* = 50.43438e^−0.03142t^	0.03142	0.96527	22.06
Sterile WSSs +free cells	*C_t_* = 52.91691e^−0.17745t^	0.17745	0.95852	3.91
Nonsterile WSSs + free cells	*C_t_* = 49.45648e^−0.14705t^	0.14705	0.91153	4.71
Sterile WSSs + immobilized A-1 cell	*C_t_* = 52.51469e^−0.21653t^	0.21653	0.98433	3.20
Nonsterile WSSs + immobilized A-1 cell	*C_t_* = 50.83235e^−0.14804t^	0.14804	0.95793	4.68

Note: *Ct* refers to glyphosate degradation (mg/L); *k* refers to degradation constant (day^−1^); *t* refers to degradation times (days); *R*^2^ refers to the correlation coefficient.

## Data Availability

Data are contained within the article.

## Supplementary Materials

The following supporting information can be downloaded at https://www.mdpi.com/article/10.3390/microorganisms13040802/s1, Figure S1: Normal plot of residuals; Figure S2: Relationship between actual and predicted removal rates; Figure S3: Relationship between predicted removal rates and externally studentized residuals; Table S1: The physiological and biochemical characteristics of strain A-1; Table S2: Effects of substrate concentration on the DIF degradation capacity of strain A-1; Table S3: Effects of inoculum on the DIF degradation capacity of strain A-1; Table S4: Effects of pH on the DIF degradation capacity of strain A-1; Table S5: Kinetic parameters for the degradation of difenoconazole at different initial concentrations by strain A-1.

## Abbreviations

The following abbreviations are used in this manuscript:

ANOVA	Analysis of variance
DIF	Difenoconazole
HPLC	High-performance liquid chromatography
LB	Luria-Bertani
MSM	Minimum salt medium
PCR	Polymerase chain reaction
QNC	Quinoline acid
rDNA	Ribosomal DNA
RSM	Response surface methodology
SA	Sodium alginate
SEM	Scanning electron microscope
SM2	Sulfamethazine
WSS	Water–sediment system
