# Supplementary material for: Immobilization of Acinetobacter sp. A-1 and Applicability in Removal of Difenoconazole from Water–Sediment Systems
Source: Microorganisms. 2025 Apr 1;13(4):802. doi: 10.3390/microorganisms13040802 (PMC12029691; doi:10.3390/microorganisms13040802)
Supplement: Supplementary file 1 [file microorganisms-13-00802-s001.zip › microorganisms-3551425-supplementary.pdf]

Supporting Information

Supplementary Table

Table S1. The physiological and biochemical characteristics of strain A-1

Characteristics	Results	Characteristics	Results
Gram staining	Negative	Lactose utilization	Positive
Oxidase	Negative	D-glucose utilization	Positive
Catalase	Positive	Citrate utilization	Negative
Gelatin liquefaction	Positive	Maltose utilization	Negative
Penicillin	Positive	Ornithine decarboxylase	Negative
Indole production	Negative	Arginine decarboxylase	Negative
H_2S production	Negative	Lysine decarboxylase	Negative

Table S2. Effects of Substrate concentration on the DIF Degradation Capacity of Strain A-1

Substrate concentration / (mg/L)	Group	Degratation of difenoconazole / (%)	Average value / (%)
	1	34.72	
**30**	2	20.89	33.12
	3	43.76	
	1	64.83	
**40**	2	70.15	65.98
	3	62.95	
	1	70.22	
**50**	2	70.22	68.63
	3	65.45	
	1	67.96	
**60**	2	66.28	67.47
	3	68.17	
	1	65.41	
**70**	2	62.69	65.51
	3	68.42	

Table S3. Effects of Inoculum on the DIF Degradation Capacity of Strain A-1

Inoculum / (%)	Group	Degratation of difenoconazole / (%)	Average value / (%)
	1	62.26	
**1**	2	69.47	65.93
	3	66.06	
	1	76.78	
**2**	2	75.71	75.43
	3	73.81	
	1	70.54	
**5**	2	67.63	69.10
	3	70.13	
	1	63.11	
**10**	2	57.8	58.45
	3	54.44	
	1	57.02	
**15**	2	51.26	57
	3	62.72	

Table S4. Effects of pH on the DIF Degradation Capacity of Strain A-1

pH	Group	Degratation of difenoconazole / (%)	Average value / (%)
	1	55.58	
**4**	2	39.76	46.12
	3	43.02	
	1	61.38	
**5**	2	62.78	64.82
	3	70.31	
	1	77.52	
**6**	2	66.42	68.50
	3	61.57	
	1	78.61	
**7**	2	85.76	82.88
	3	84.28	
	1	73.38	
**8**	2	69.86	71.68
	3	71.81	

Table S5. Kinetic parameters for the degradation of difenoconazole at different initial concentrations by strain A-1

Difenoconazole concentration / (mg/L)	Regression equation	k / (day^{-1})	R^2	$t_{1/2}$/ (days)
**30**	$C_t = 21.30746e^{-0.03196t}$	0.03196	0.98794	21.68
**40**	$C_t = 22.04618e^{-0.04287t}$	0.04287	0.87842	16.17
**50**	$C_t = 16.80561e^{-0.02542t}$	0.02542	0.93184	27.28
**60**	$C_t = 24.48607e^{-0.02784t}$	0.02784	0.97189	24.90
**70**	$C_t = 30.69394e^{-0.02074t}$	0.02074	0.98712	33.42

Note: C_t refers to glyphosate degradation (mg/L); k refers to degradation constant (day^{-1}); t refers to degradation times (days); R^2 refers to the correlation coefficient.

Supplementary Figure

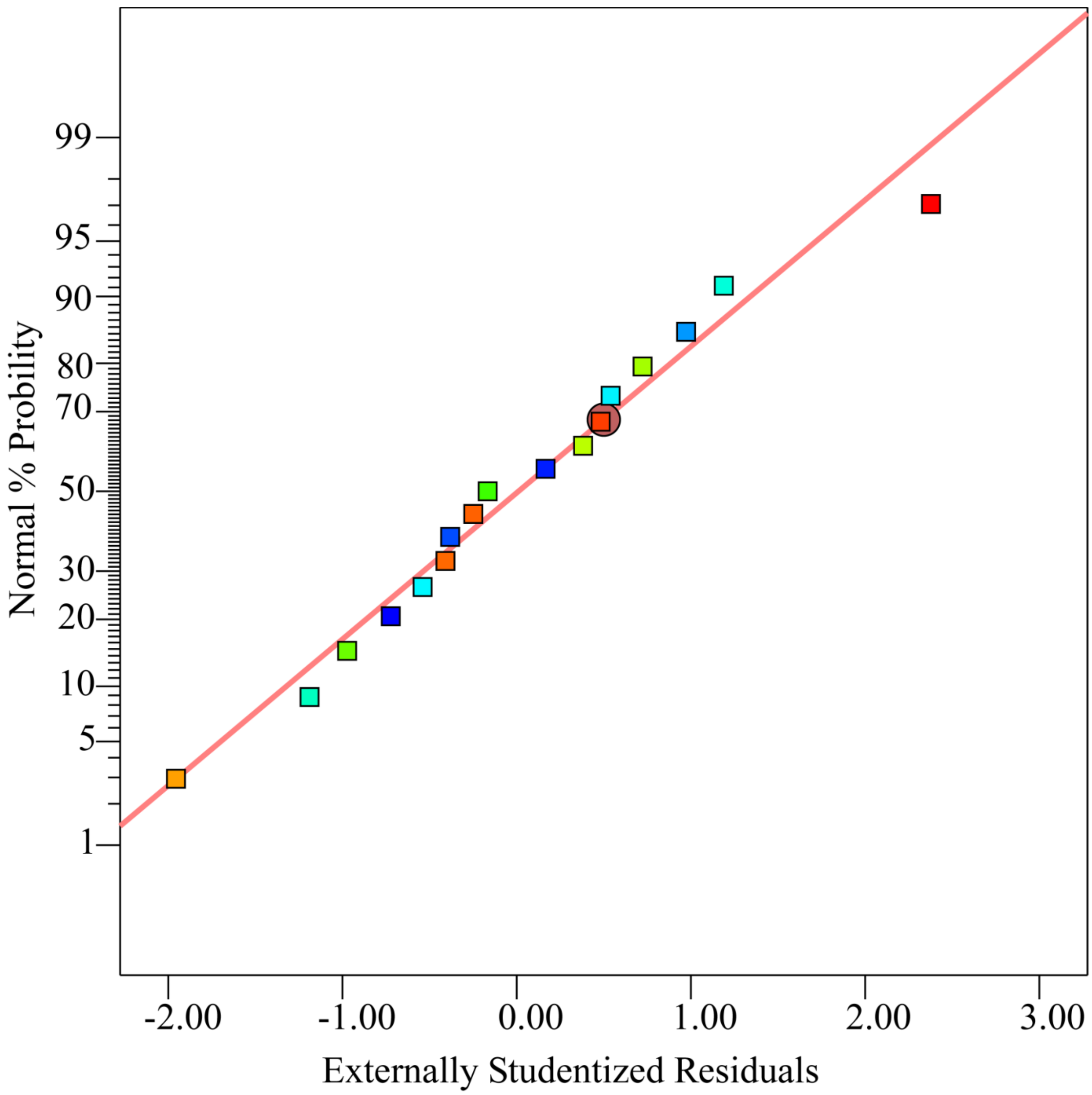

Figure S1 Normal plot of residuals.

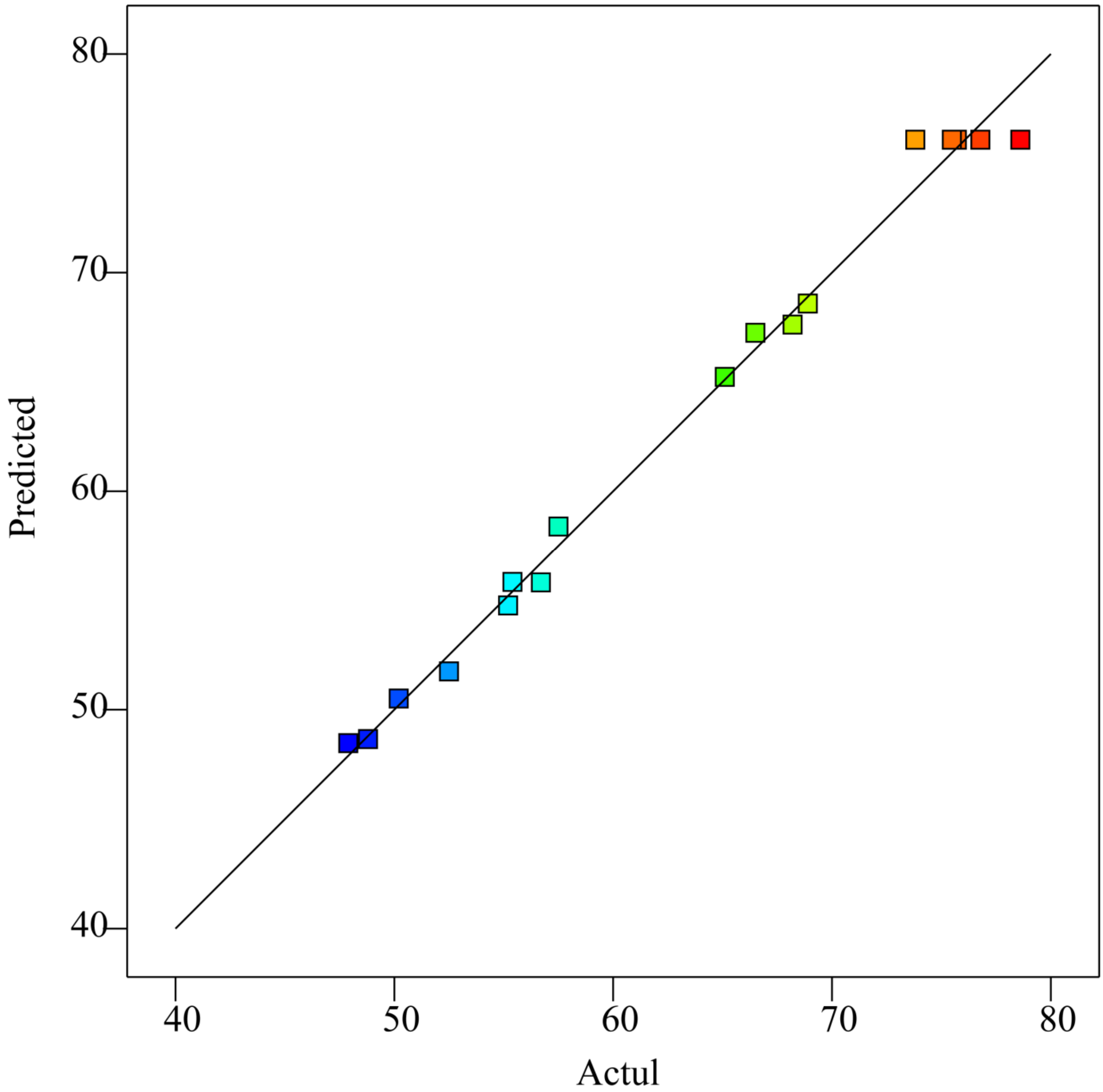

Figure S2.Relationship between actual and predicted removal rates.

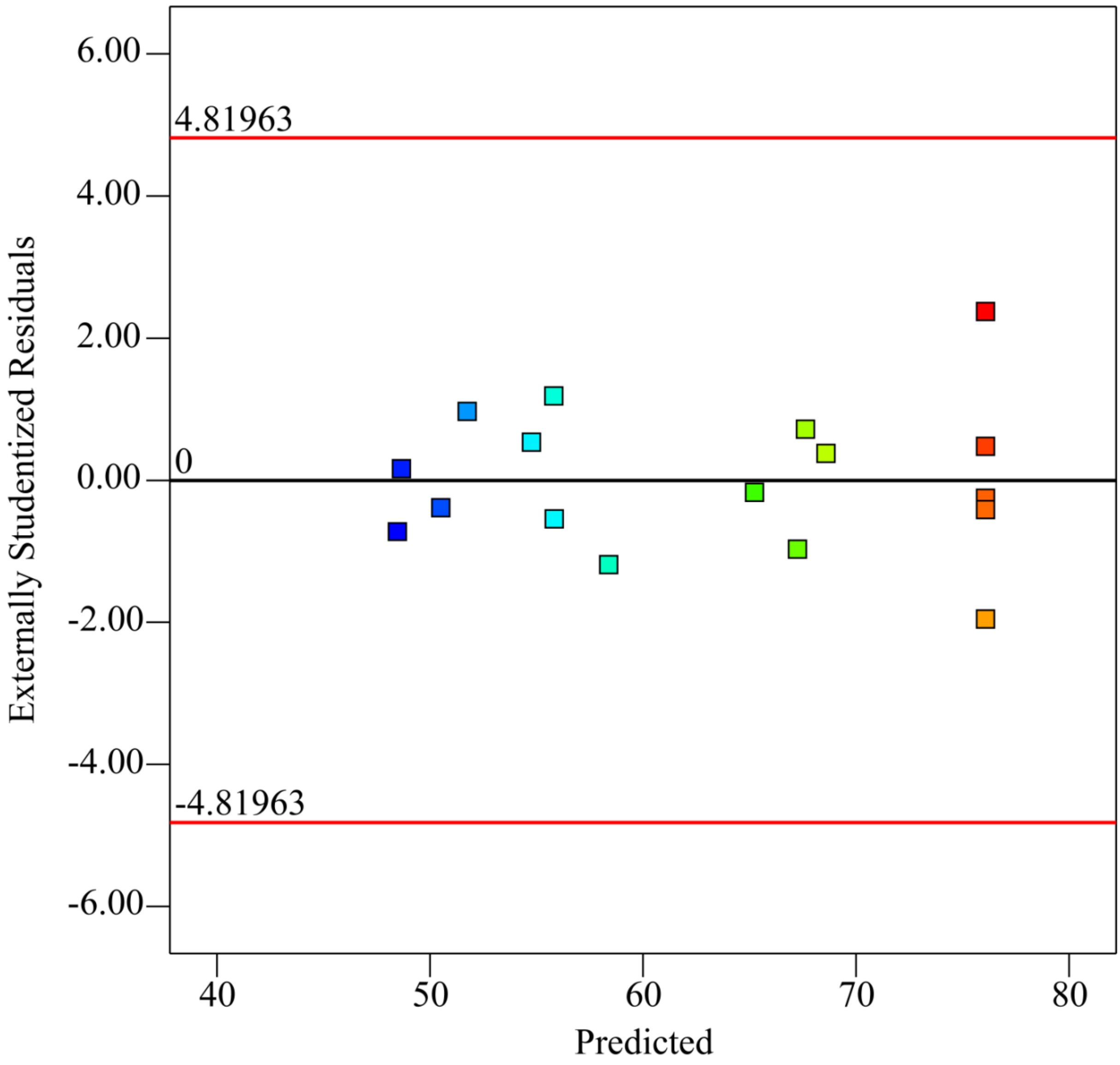

Figure S3 Relationship between predicted removal rates and externally studentized residuals.
